# Looking into the Eyes—In Vitro Models for Ocular Research

**DOI:** 10.3390/ijms23169158

**Published:** 2022-08-15

**Authors:** Krystyna Lieto, Rafał Skopek, Aneta Lewicka, Marta Stelmasiak, Emilia Klimaszewska, Arthur Zelent, Łukasz Szymański, Sławomir Lewicki

**Affiliations:** 1 Department of Regenerative Medicine, Military Institute of Hygiene and Epidemiology, Kozielska 4, 01-163 Warsaw, Poland; 2 Department of Molecular Biology, Institute of Genetics and Animal Biotechnology, Polish Academy of Sciences, Postępu 36A, 05-552 Jastrzębiec, Poland; 3 Military Centre of Preventive Medicine Modlin, 05-100 Nowy Dwór Mazowiecki, Poland; 4 Department of Medicine, Faculty of Medical Sciences and Health Sciences, Kazimierz Pulaski University of Technology and Humanities in Radom, 26-600 Radom, Poland; 5 Department of Cosmetology, Faculty of Medical Sciences and Health Sciences, Kazimierz Pulaski University of Technology and Humanities in Radom, 26-600 Radom, Poland

**Keywords:** in vitro eye models, 3D eye models, tissue engineering, ocular toxicity, eye irritation, corneal equivalents

## Abstract

Animal research undoubtedly provides scientists with virtually unlimited data but inflicts pain and suffering on animals. Currently, legislators and scientists alike are promoting alternative in vitro approaches allowing for an accurate evaluation of processes occurring in the body without animal sacrifice. Historically, one of the most infamous animal tests is the Draize test, mainly performed on rabbits. Even though this test was considered the gold standard for around 50 years, the Draize test fails to mimic human response mainly due to human and rabbit eye physiological differences. Therefore, many alternative assays were developed to evaluate ocular toxicity and drug effectiveness accurately. Here we review recent achievements in tissue engineering of in vitro 2D, 2.5D, 3D, organoid and organ-on-chip ocular models, as well as in vivo and ex vivo models in terms of their advantages and limitations.

## 1. Introduction

The number of factors that can damage human tissues increases every year. For example, smog, substances contained in cosmetics, unnatural food additives, and UV radiation have a harmful effect on our skin and eyes. Moreover, every year industry delivers thousands of new chemical substances which are necessary for new medicines, chemicals, or food additives. Therefore, biocompatibility assessments of each new compound, especially one that involves animal testing, is impossible. Moreover, the number of studies performed on animals has to be limited in Europe by the law (i.e., Directive 2010/63/EU).

Optic neuropathies, such as glaucoma, anterior ischaemic optic neuropathy (AION), traumatic optic neuropathies, optic neuritis, etc., need new treatment options, which in turn require the development of disease models [1]. However, the pathophysiological mechanisms of these diseases are not fully understood; therefore, developing an animal model is a tough challenge. Moreover, due to the physiological differences, animal models differ significantly from human diseases [2]. For example, rodents’ eyes do not have maculae or foveae, and 85–90% of their optic nerve axons decussate to the other side of the brain [1]. On the other hand, monkeys’ anatomy of the retina and optic nerve is almost identical to that of human eyes. Still, monkey breeding is complicated, very expensive, and time-consuming; therefore, the number of tests performed on individual animals is limited. As a result, monkeys are often used in the stage just before clinical trials on humans [1].

Historically, one of the most popular experiments performed on animal eyes is the Draize test. Developed in 1944 by American toxicologists John H. Draize and Jacob M. Spines, it was widely used to study cosmetics and other chemicals. However, the test arouses many controversies due to the lack of reliable and objective results. In fact, the test was never correctly validated. Briefly, the test is based on applying the test substance directly to the eye, but the exposition time is not well defined. After observing the eye reaction for some time, the substance is washed from the eye, and the animal is observed for another two weeks. The result is subjectively assessed by the operator [3]. Moreover, the test is considered incorrect mainly due to anatomical and biochemical differences between the human and the animal (mostly the rabbit) eye. Therefore, currently, the Draize test is not performed. Instead, chemicals are usually tested using the EpiOcular eye irritation test, in vitro cytotoxicity assay, and irritation tests on the rabbit’s skin. Each of these tests has advantages and disadvantages, but none of them allows for multifactorial compound-eye interaction evaluation. The EpiOcular eye irritation test is an in vitro alternative which allows for the assessment of acute eye irritation in response to the topical administration of chemicals onto the EpiOcular cornea epithelial model. The test makes cytotoxic effect measurement possible and provides a tool for eye-hazardous chemical identification.

For these reasons, it is crucial to develop new in vitro tissue models to study all substances for ocular treatment and to understand the development and molecular causes of eye diseases [4]. Here, we summarized all cellular and tissue-specific animal models used in in vitro eye studies.

## 2. Eye Structure

The eye is a highly complex biological machine (Table 1). The human eye has the shape of a sphere about 24 mm in diameter. It is filled with a vitreous body that allows the shape of the ball to be maintained. It is located in the eye socket, which reduces the risk of mechanical damage. The eye is divided into two parts: external and internal. The eye’s outside layers are tough, elastic structures, with a white sclera and transparent cornea which provide eye shape [5]. The second inner layer is the vascular membrane, including the iris, ciliary body, and choroid. The third layer is the retina. It consists of light receptors, cells, nerve fibers, and blood vessels originating from the central retinal artery [6]. The primary function of the eye is to convert light pulses into electrical signals, which are transmitted to the brain and converted into images. Light is refracted by the cornea and the lens, which results in a sharp, inverted, reduced image formed on the retina. The amount of light reaching the receptors is regulated by the iris, which changes pupil diameter [6].

The retina in vertebrates is characterized by light-sensitive structures and covers 60% of the back of the eyeball. It is located above the choroid. It consists of 10 cell layers and contains photoreceptors called rod cells and cone cells [7]. These cells contain a visual pigment which is located in the cell membrane. Rod cells allow the recognition of shapes and motion at low light intensity [8]. Cone cells are responsible for seeing color and detail in a more intense light than rod cells. Individual cone cells differ in sensitivity at wavelength, which allows us to distinguish colors. In humans, cone cells are concentrated in the macula—a small pocket of the retina center. The optic nerve’s axons that leave the retina at the ONH form a blind spot. There are no photoreceptors in that spot [9].

The eyeball is surrounded by the connective tissue—the sclera. It is a hard layer protecting the inner structures of the eye. In addition, it stiffens the eyeball like a bag of collagen and elastic fibers [10]. The sclera is thinner, more permeable to light, and creates a transparent cornea on the front. It refracts the light rays so that they fall on the lens. It is susceptible to pain and able to partially regenerate. It is avascular and consists of six layers (epithelial, Bowmann, stroma, Dua’s, Descemet, and endothelial).

The conjunctiva lines the inner surface of the eyelid. It contains a lot of mucous cells, which ensures the constant humidity of the eyeball—it produces mucus and tears. It covers the eyeball up to the edge of the cornea. It has a very high regeneration ability. The conjunctiva is sensitive to any irritation, such as smoke, dust, or chemical substances. These factors can lead to conjunctivitis. During this type of inflammation, blood vessels are firmly filled with blood, causing redness and swelling of the eye [11]. Glands are dispersed in the conjunctiva, and the lids secrete mucus, water, and lipids, forming a tear film whose primary function is to moisturize and cleanse the eye from undesired foreign bodies if needed. There are many glands dispersed in the conjunctiva and the lids that secrete mucus, water, and lipids, thus forming the tear film. However, the main lacrimal gland (responsible for emotional tears) is outside the eye structure.

Even though the eye is a very specialized organ, there is significant progress in the development of tissue engineering, and newer and more suitable in vitro models are emerging. The search for such models is caused, among others, by increasing awareness of the welfare of animals used in experiments, including toxicological effects [12].

## 3. In Vitro Ocular Models

**Figure 1:**
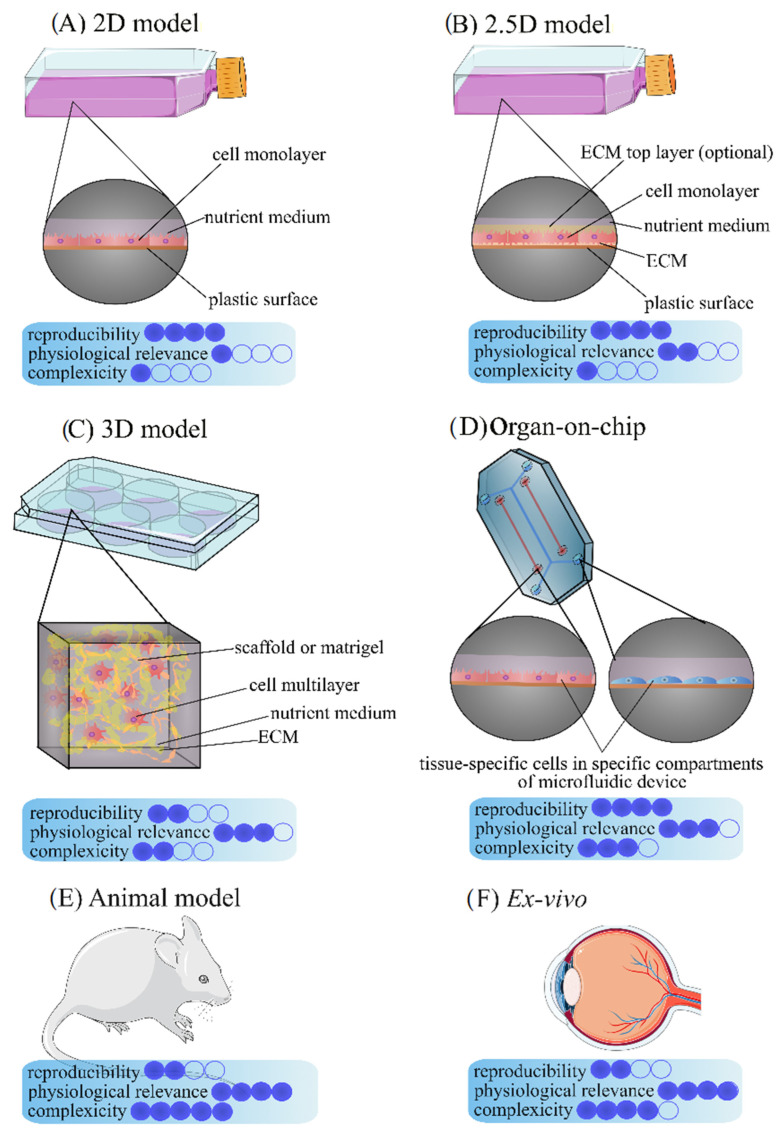
Schematic diagram of of eye models types. The figure was created using SMART (Servier Medical ART) modified graphics, licensed under a Creative Commons Attribution 3.0. Generic License.

**Figure 2:**
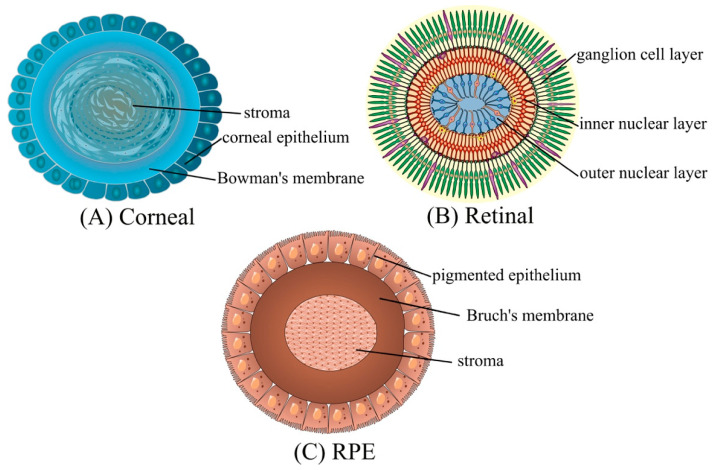
Schematic diagram of eye organoids. The figure was created using SMART (Servier Medical ART) modified graphics, licensed under a Creative Commons Attribution 3.0. Generic License.

Over the years, many alternative assays were developed to accurately evaluate ocular toxicity and drug effectiveness. Here we present recent achievements in tissue engineering of various ocular models in terms of their advantages and limitations (see also Figure 1 and Figure 2). 

### 3.1. 2D Eye Models

Currently, 2D models are the most popular ones in ocular research. Two-dimensional cell line culture is an inexpensive, well-established model providing results that are easy to compare with the vast literature. However, the unquestionable drawback of these culture systems is the lack of predictivity in research connected with the fact that cells growing on a flat surface are not an equal representation of the cell environment in the organism.

#### 3.1.1. Pigment Epithelium Cell Lines

One of the most common 2D models is immortalized retinal pigment epithelium (RPE) cell lines. Primary cultures of retinal cells are challenging to handle. Obtaining a homogenous cell line that is not contaminated with other eye cells is challenging. Furthermore, isolated cells often quickly change their properties. For example, cells can lose keratin-containing intermediate filaments [13]. Cell transformation using the SV40 virus managed to obtain a line that retains the characteristics of retinal cells [14]. These cells are characterized by appropriate polarization and monocellular epithelial cell formation.

In 1995, RPE cells were first isolated by Davis et al. from a patient. However, the RPE cell line was only used in toxicity tests because the cells lost the characteristics of normal metabolism, adequate cytoskeleton polarization, and enzyme activity [14]. In the literature, primary models of RPE cell culture obtained from mice (i.e., Mouse Retinal Pigment Epithelial Cells-Hpv16 E6/E7, Immortalized) [15], rats (RPE primary cells isolated from PVG rats susceptible to experimental uveitis development; RPE isolated from Long Evans rats) [16,17], chickens (primary RPE cells isolated from domestic chickens embryos at stages 29–31 of development) [18], bovines (primary RPE cells) [19], and frogs (Xenopus laevis isolated primary RPE cells) [20] have also been described.

The human cell line ARPE-19 has structural and functional properties characteristic of RPE cells in vivo (in rats, RPE-J) [11]. This line is essential because the number of tissue donors is limited [21]. Studies on ARPE19 showed several features confirming the usefulness of this line for retinal pigment epithelial examination, such as expression of characteristic RPE cell markers, CRALBP, and RPE65, secretion of IL-6 and IL-8, as well as morphological polarization in monolayers, and ability form tight-junctions [22,23].

RPE-340 are primary cells isolated from humans which have epithelial morphology, but after several passages, their ability to replicate is limited [24]. Human RPE cells are good models for pharmacodynamic and physiological evaluation of a drug’s effect on the choroid-RPE-photoreceptor, but after 40–60 population doublings, they go into a senescence state [24]. To develop a cell line with an extended lifespan, RPE-340 was transfected with a plasmid expressing the human telomerase reverse transcriptase subunit (hTERT), creating a new cell line—hTERT-RPE-1 (human retinal pigment epithelial RPE-1) [25]. This way, the lifespan of hTERT-RPE-1 is extended without any alterations in the population, doubling time and RPE-340 characteristic features [26]. hTERT-RPE-1 is reported to be an excellent model for epigenetic regulation studies [27,28,29]. Unfortunately, this line still has its limitations. The handling lasts 20 passages longer than RPE-340, but after this period, the cells change their morphology and function under the phenomenon called deadaptation [30,31]. Due to the low availability of primary human RPE cultures, validating and comparing this cell line with immortalized cell lines is challenging. The perfect line of human RPEs has yet to be developed.

The R28 immortalized retinal precursor cell line originating from postnatal day 6 rat retinal culture has been frequently used in in vitro and in vivo studies [32]. R28 provides an important system for understanding retinal cell behavior aspects such as differentiation, cytotoxicity, light stimulation, and neuroprotection. Although R28 originated from single clones, they remained highly heterogenous, suggesting the precursor character of these cells [32]. This cell model has been used in various toxicity experiments in vitro [33,34,35,36,37]. In addition, R28 exerts a high potential for studying the neuroprotective properties of chemical compounds [38,39,40]. Latanoprost was one of the drugs validated on R28 under the angle of cytoprotective properties [33].

RGC-5 was previously described as a rat-derived, transformed retinal ganglion cell line and is widely used in glaucoma research [41]. After more than 220 published papers worldwide involving the use of the RGC-5 cell line, it was reported that these cells are in fact 661W, a mouse SV-40 T antigen transformed photoreceptor cell [41,42]. The 661W cell line was present in the laboratory of origin of RGC-5; therefore, the most probable scenario was the cross-contamination of the newly developed cell line with 661W. This incident has shown how crucial the proper culture protocols and DNA profiling of newly-developed cell lines are. 661W is a model of cone photoreceptor cells. This cell line was widely used as a model for research on macular degeneration, but studying retinal ciliopathies such as retinitis pigmentosa is believed to be possible [43]. 661W shows properties of both retinal ganglion and photoreceptor cells, providing a functional photoreceptor model [43,44]. Moreover, 661W are believed to be an alternative model to the hTERT-RPE-1 cell line previously used for small molecule screening to identify new treatments for retinal ciliopathies [45]. 661W shows potential in studying ciliopathy disease genes not expressed or expressed at a low level in hTERT-RPE-1 cells [43].

#### 3.1.2. Cornea Cells

The primary cornea cultures on which the individual in vitro models were developed come mainly from rabbits. Rabbit corneal epithelial cells (RbCEpC) help assess drug safety, pharmaceutical effects, corneal development, pathology, glaucoma, viral infections, keratitis, ocular hypertension, and even special contact lenses that provide sustained, extended-release of ophthalmic drugs [31]. Human corneal epithelial cells (HCEpC) have been used as models for studying corneal damage and reconstruction, re-epithelialization of the eye following surgery, and the effects of degradative enzymes. Corneal research mainly focuses on developing a model of drug permeation through this structure [46]. The models of corneal culture used in cellular research concern simple monolayers, the multilamellar epithelium, and very complex three-dimensional (3D) tissues resembling the functional cornea. HCEpC was used to create a single cellular layer later used for transplantation [47].

Several commercially available in vitro cornea models are destined to be cultured in 2D models (monolayers). One such model is HCE-T, in which cells are grown on the collagen membrane and are located at the air-liquid interface with the serum-free medium. The cells have the features of the primary cell line and form a stratified epithelium whose morphology can be modulated with calcium. Moreover, the cells expressed specific corneal epithelial cell markers such as epidermal growth factor (EGF), EGF receptor, basic fibroblast growth factor (basic FGF), transforming growth factor-beta 1 (TGF-beta 1), and interleukin-1 alpha (IL-1 alpha) [48]. 

#### 3.1.3. Corneal Endothelial Cells

The role of corneal endothelial cells (CECs) is to control corneal transparency. Unfortunately, the cells exhibit limited proliferative capability; therefore, their dysfunction may be one of the causes of blindness. One of the gold standards in treatment of corneal endothelial dysfunction is the donor isolated corneal transplant [49]. The first culture of human corneal endothelial cells from donors was established by Pistov et al. in 1988 [50], and from that time, plenty of protocols and newly designed biomaterials for the propagation of these cells were developed [51]. However, due to the low proliferation rate of primary CECs culture, protocols for immortalized cells were established [52,53]. Currently, several immortalized human CECs are available in the market and are used mainly to understand corneal endothelial cell dysfunctions. Two clonal cell lines derived from the immortalization of human corneal endothelial cells (obtained from the donor) were described by Valtink et al. [54]: B4G12 and H9C1 cells. B4G12 cells are polygonal, strongly adherent cells, which form a strict monolayer and H9C1 cells are less adherent and formed floating spheres. Both cell lines exhibited the characteristic expression of corneal endothelial cell markers; however, on different levels. Therefore, the authors concluded that the B4G12 cell line is a good model of differentiated CECs, and H9C1 is a good model for developing or transitional CECs. Alternatives for donor corneal endothelial cells or cornea endothelial cell lines may be pluripotent stem cell-derived corneal endothelial cells. The cells generated from a cryopreserved human embryonic stem cell (hESC) are stable, express corneal endothelial cell markers, and have an improved proliferation rate compared to primary CECs [55,56]. 

#### 3.1.4. Conjunctival Cells

The most popular eye conjunctival test model is the rabbit conjunctiva. For the first time in 1996, Saha et al. isolated the primary culture of the rabbit’s conjunctiva. The model created by him represents a tight epithelial barrier [57]. This model is constantly being improved. The most significant difficulty was adapting the cells of this model to contact with air, just like in the natural eye. The use of additional filters (for example, the Transwell filter) allowed cell growth at the air-liquid interface. The layers of the conjunctival epithelial cells showed transepithelial resistance and a difference in potential [58]. The conjunctival epithelial cells are polygonal with many microvilli [59]. The primary culture of conjunctival cells was also obtained from bovines [59] and rats. The immortalized rat conjunctival (CJ4.1A) cell line was created by transfection of SV 40 [60]. CJ4.1A expresses the SV40 T antigen, conjugal cytokeratin 4, and cytokeratin specific for goblet cells 7, but not the cytokeratin 12. The line’s lifespan is very long—line cells can be cultured for over 60 passages, and the population doubling times were 22 ± 7 h [60].

The development of methods for obtaining the primary culture of conjunctival cells has contributed to the development of transplantation techniques for heterotopic or allogeneic grafts. After severe damage to the conjunctiva, it is possible to restore its function by taking a piece of epithelium from a healthy eye, multiplying it in a cell culture, and implanting it in the affected conjunctiva [61]. Besides the primary cell culture, several established conjunctiva cell culture lines also exist. An example would be two human immortalized conjunctival cell lines: HCjE [62] and IOBA-NHC [63]. These cells have a typical epithelial morphology of the human epithelium, and after exposure of the cells to inflammatory mediators (IFNγ and/or TNFα), they increase the expression of the intercellular adhesion molecule (ICAM)-1 and MHC class II cell surface receptor (HLA-DR) [63].

The above-mentioned 2D models have their advantages but also their limitations. Firstly, these models are exceptionally delicate, and their manipulation must be meticulous. For example, the layer is easily damaged and dried. In addition, the models do not take into account cell to cell communication and the influence of immunological factors, which probably have a tremendous impact on the regeneration of this structure. Finally, based on these models’ results, it is impossible to recapitulate all the processes occurring in the cornea in the human eye [64]. Therefore, the researchers decided to develop more complex, multicellular eye models.

### 3.2. 3D Models

Three-dimensional models better replicate the organism-environment compared to two-dimensional cultures. Cells grow in every dimension and closely replicate tissue in vitro, which complements 2D cell culture [65]. Although 3D models provide us with more information than 2D models, they are more challenging to handle. Multilayer models respond to more and more questions about corneal damage and disfigurement, but they are still far from the complex equipment that the eye is. For example, they lack the lacrimal apparatus responsible for cleansing and supporting regeneration [66]. Few 3D cornea models have been developed to this point.

EpiOcular™, developed in 2010, was obtained from cultured human epithelial cells. The cells showed a morphology and expression of biomarkers similar to the intact human cornea and maintained its thickness and permeability [67]. The EpiOcular model was used to assess the eye irritation potential of surfactant and surfactant-based formulations. Based on the protocol, the compound is considered to be an irritant when more than 50% of the cell die as compared to the negative control [68,69]. This test is validated and under review by the European Center for the Validation of Alternative Methods (ECVAM).

Clonetics (cHCEC) was developed in 2011 and was obtained from human corneal epithelial cells. Research using the model provides information on the assessment of corneal penetration by various chemical compounds (e.g., ophthalmic drugs) [68]. cHCEC was examined by RT-PCR for the expression profile of drug-metabolizing enzymes (e.g., CYP P450s and UGT1A1) and transporters in cHCE in comparison to the human cornea [70].

The SkinEthic (HCE) 3D cornea model comprises immortalized human mucosa cells; cells are grown at the air-liquid interface using a polycarbonate membrane. Under appropriate conditions, the cells differentiate and form the three-dimensional (3D) stratified epithelium and have non-keratin structures [69]. The advantage of the model is the possibility of administering dissolved substances in organic and inorganic solvents at any concentration (a very concentrated solution or minimal drug application concentrations can be applied) and the preservation of conditions similar to the eye mucosa of the human eye. Immortalized cells are grown in a dedicated medium and form a histologically multilayered construct with a thickness of 60 μm. The HCE secretes the same mucins found in the human cornea in vivo and expresses CD44 and keratin. This model is used to study phototoxicity, irritation, corrosivity, and the transport of substances [71,72].

The LabCyte CORNEA-MODEL is produced from normal human cornea epithelial cells [73]. It was developed by differentiating and stratifying cornea epithelial cells and is meant to be used to identify irritant chemicals in eye irritation tests. The corneal epithelial cells are cultivated on an inert filter substrate for 13 days with a medium containing 5% FBS. Proliferating cells build up in a multilayer structure consisting of a fully differentiated epithelium with features of the average human corneal epithelial tissue [74].

The limited source of corneal tissue to form a 3D-model, the short-lived life cycle of the corneal cells themselves, and the time-consuming culture contribute to problems with the industrialization of culture. The use of many commercially available 3D models was limited by the rapid differentiation of cells leading to problems with maintaining cell culture [75]. Many attempts have been made to increase the in vitro culture cycle of corneal epithelial cells concerning telomerase reverse transcription gene transfection, viral transfection, and the induction of spontaneous mutations. Nevertheless, the abnormal phenotype of these cells, which can lead to the potential risk of tumorigenesis, is not desired in the construction of new cornea models. Therefore, Li et al. enriched cornea cells with limbal stem cells providing additional expansion and development stimulation [75]. The addition of limbal stem cells promoted development and cell expansion. Moreover, it enabled the large-scale production of a new 3D model. Use of the corneal stromal layer of the animal to stimulate a specific microenvironment for limbal stem cells resulted in their differentiation into cornea epithelial cells.

Zuguo et al. proposed a new in vitro xeropthalmia model by dissecting the conjunctival epithelium and subconjunctival matrix, culturing it on a collagen I coated dish submerged in a culture medium. The in vitro dry eye model is obtained after 4–20 days. The invention can be used to research dry eye squamous metaplasia, ocular surface epithelial barrier damage, epithelial mucin change, to test new drugs, or to find new methods for dry eye treatment [76].

The model created by Minami et al. consists of bovine epithelial, stromal, and endothelial cells in a collagen gel matrix. The epithelium consists of five to six layers, and the epithelial cells produce keratin, which is a fundamental multilayer model for the cornea [77]. In addition, some corneal models use cell lines from different animals. In these models, individual layers come from mice, rabbits, bovines, and pigs [65,78,79].

### 3.3. 2.5D Models

2.5D models seem to be an alternative approach compared to 2D and 3D cultures. In 2D, cells are grown on a flat surface, while 3D models are based on cells embedded in an extracellular matrix (ECM) and/or scaffolds that provide a proper three-dimensional environment. In 2.5D cultures, cells are grown in an extracellular matrix (ECM) layer which often is not flat but unregular with projections and grooves, thus, providing an intermediate between 2D and 3D conditions [80]. 

### 3.4. Ex Vivo Models

One alternative to the Draize test is harvesting organs for examination from animals used for meat (ex vivo model). Eyeballs are isolated from bovines (BCOP), rabbits (IRE), pigs (PCOP), and chickens (ICE). This test was accepted internationally in 2009 and is used to research if significant tissue damage can occur [81]. Tests on the models mentioned above are based mainly on histological and light transmittance through cornea analysis. The pigs’ cornea provides the highest degree of similarity to the human cornea, especially in tests involving substances dissolved in water [82]. Unfortunately, all the models mentioned above have serious drawbacks, primarily resulting from anatomical differences. In addition, these models can only be used to study individual eye structures. Therefore, they do not allow for general-purpose research. It is vital to create cell microenvironments that support tissue differentiation and changes, tissue-tissue communication, and spatiotemporal chemical and mechanical gradients of the microenvironment of living organs [83].

Yu F. proposed an ex vivo mammalian cornea culture system used for chemical tests of consumer products [84]. This system closely resembles in vivo testing by maintaining the corneal structure, architecture, and epithelial cell interaction. The cornea or the whole eye is excised and placed on an agar or collagen scaffold. It is then submerged in a culture medium until the medium covers the limbus. The upper part of the cornea is not submerged in medium. The tested reagent is administered directly to the cornea. The inventor claims that the system may be used to replace the use of Draize’s test in many situations. This system allows drug testing without using live animals. The corneas or eyeballs may be, for example, easily acquired when dissecting rabbits for meat or fur industry purposes.

### 3.5. Spheroids, Organoids, and Organ-on-Chips

New techniques and technologies in cell culture allow the development of more proper and scientific-useful models for ocular research. Here we described three types of it: spheroids, organoids, and organ-on-chips, a summary of which is presented in Table 2.

#### 3.5.1. Spheroids

Spheroids are self-assembly aggregated cells that spontaneously organize themselves into spherical-shaped structures. This phenomenon occurs naturally during embryogenesis, morphogenesis, or organogenesis. In in vitro culture, single cells may constitute multicellular spheroids after applying appropriate cell culture techniques (i.e., pellet culture, the hanging drop method, culture in the extracellular matrix, or others) [85]. Spheroids may have a different biological response to various factors due to the presence of a concentration gradient of nutrients, oxygen, or metabolites between cells from the outside and the inside part of the spheroid. Spheroids are mainly used in cancer research [86,87]. However, the technique of 3D multicellular culture with spheroids is also used in cellular research. Lu et al., using air-lifting 3D spheroid formation techniques, developed an in vitro model for research on the ocular surface and tear film systems. The model was composed of rabbit conjunctival epithelium and lacrimal gland cell spheroids [88]. The model allowed for the creation of the aqueous and mucin layers of the tear film, which may facilitate research on dry eye. A Japanese-German research group generated multicellular spheroids from human-donor RPE cells cultured in a methylcellulose matrix [89,90]. The model mimics the in vitro drusen model, which might help understand the pathogenesis of drusen-related diseases such as AMD. Sherwin’s group from New Zeeland developed methods for isolation and propagation of spheroid human peripheral cornea using a clear cornea component of the rim isolated from a donor [91,92]. They found that generated spheroids implanted into frozen-stored corneoscleral tissue worked as limbal stem cell centers and proliferated to reproduce limbal cells. Spheroids are also used in ocular cancer research. There are several spheroid models of retinoblastoma (cells isolated from human intraocular tumors) which are used to develop new cancer treatments [93] or to understand retinoblastoma pathophysiology [94,95]. 

#### 3.5.2. Organoids

Organoids are stem cell derived 3D structures with organ-level functions. They are composed of self-organizing organ-specific cells derived from embryonic stem cells, induced pluripotent stem cells, or organ-restricted adult stem cells [96,97].

One of the most well-known ocular organoid models is a model described by Eiraku et al. [98]. The authors used mouse embryonic stem cells and show that ESCs in differentiation medium are self-organizing into optic-cups in 3D culture. Susaimanickam et al. developed an organoid model based on human embryonic stem cells (ESCs) or human induced pluripotent stem cells (iPSCs) cultured in a retinal differentiation medium supplemented with noggin [99]. The addition of noggin is crucial because of the protein’s (a BMP inhibitor) involvement in the retinal differentiation of pluripotent stem cells during embryonic and organoid development. After two weeks, the culture gave rise to retinal and corneal primordia, and after six to eight weeks, primordia developed into minicorneas with specific morphological and marker similarities to the human cornea. This model may be used in basic research and regenerative applications. In addition, the use of organoid models with different ranges of time culture could provide us with data regarding drug toxicity in different stages of eye development. A congruous model or cornea organoids was developed by Foster et al. [100]. In this model of the cornea, three distinct cell types with the expression of key epithelial, stromal and endothelial cell markers were obtained. Mellough et al. in 2012 showed that ESC and iPSC cultured in ventral neural induction media (VNIM) supplemented with noggin, Dickkopf-1, Insulin-like growth factor 1, Lefty A, Human Sonic Hedgehog, and 3, 30, 5-triiodo-L-thyronine may develop retinal photoreceptor cells [101]. Later, they showed that VNIM can differentiate both EPS and iPSC cells, but the presence of IGF-1 is essential for the development of 3D ocular-like structures containing retinal pigmented epithelium, neural retina, primitive lens, and corneal-like structures [102]. In the latest work, Mellough et al. found that different embryoid bodies’ (EBs) generation protocols affect the method and maintenance conditions that determine the later differentiation and maturation of retinal organoids [103]. The generation of more advanced in vitro multiocular organoids from human iPSCs cells was proposed by Isla-Magrané et al. [104]. In this protocol, organoids are differentiated in three different media, which leads to obtaining multicellular organoids after 150 days. Firstly, 75% confluent hiPSCs were cultured on Matrigel in an induction medium (DMEM/F12, 5% fetal bovine serum, nonessential amino acids, GlutaMax, N2, B27, β-glycerol phosphate, nicotinamide, Noggin, DKK1, bFGF) for 30 days. Next, all-trans retinoic acid (ATRA) was added for the next 60 days. Finally, cells maintained in a medium with ATRA for the next 60 days develop multiocular and corneal organoids, and cells cultured without ATRA and with triiodothyronine develop retinal organoids, RPE organoids, and multiocular organoids.

Recently, the National Centre for the Replacement Refinement & Reduction of Animals in Research (NC3Rs) and The National Eye Institute established a relationship that will result in the construction of organoids for drug screening, disease modeling, and regenerative medicine [105]. Therefore, a retinal 3D model is constantly being developed to fulfill those criteria [105,106,107]. The retinal 3D model contains bioprinted Müller cells, microglia, neurons, and RPE cells [108].

#### 3.5.3. Organ-on-Chips

Organ-on-chips (OoC) are structures created by combining microfluidic technology, biomaterials, and cell culture methods [97]. Many organ-on-chips were used to research the permeability of the epithelium. Puleo et al. created a microfluidic device consisting of a bilayer structure of a corneal epithelial layer, a layer of stromal cells, and collagen vitrigel substrate [109]. Bennet et al. invented a cornea organ chip including epithelial layers, Bowman’s membrane, basement membrane, and a device simulating tear flow dynamics. The measurement of epithelium permeability underflow showed results similar to in vivo measurements [110]. Cornea and retina chips are powerful and promising in vitro tools to study drug effects and therapeutic approaches, yet the chips are still minimal and straightforward 60 [97].

Recently, Seo and Huh proposed a cornea-on-chip “human blinking eye model” [111]. The system mimicked spontaneous eye blinking in humans with keratinocytes cultured to mimic the epithelial cells and form a corneal structure. Blinking imitation was performed by integrating a tear chamber in a 3D-printed eyelid [112].

DynaMiTES’ Dynamic Micro Tissue Engineering System was developed from cornea immortalized cells. The system allowed for the measuring of transepithelial electrical resistance in real-time by implementing two electrodes into the system, providing a non-invasive way to monitor cell conditions [113].

Although organoids and organ-on-chips carry indisputable benefits, their potential in drug testing has yet to be closely examined. The main issues concerning drug assays relate to permeation and accessibility of the ocular surface of the tested models [97].

**Table 2:** Spheroids, organoids, and microphysiological models for in vitro ocular research.

**Spheroids**				
Initial cells	Targeted cells	Culture techniques	Research possibilities	References
primary rabbit conjunctival epithelial cells (CECs) and lacrimal gland (LG) cell	cells that produce the aqueous and mucin layers of the tear film	an orbital shaker than Matrigel^®^ matrix	dry eye disease	[88]
human RPE cells (hRPECs) obtained from donors	retinal pigment epithelium	culture in methylcellulose	Drusen-associated degeneration in the retina	[89,90]
human peripheral cornea	transplantable elements for limbal stem cell repopulation and limbal reconstruction	clear cornea component of the rim from a human donor	Regenerative medicine	[91,92]
human retoinoblastoma	human retoinoblastoma	different techniques	retinoblastoma	[93,94,95]
**Organoids**				
Initial cells	Targeted cells	Culture techniques	Research possibilities	References
mouse embryonic stem cell (ESCs)	mechanically rigid pigment epithelium, embryonic optic cup, stratified neural retinal tissue	G-MEM supplemented with knockout serum replacement, nonessential amino acids, pyruvate, mercaptoethanol.	development of eye, eye disorders, disease modeling,	[98]
human embryonic stem cells (ESCs) or human induced pluripotent stem cells (iPSCs)	after two weeks: retinal and corneal primordia, after six to eight weeks: primordia developed into minicorneas with specific morphological and marker similarities to the human cornea.	culture in a retinal differentiation medium supplemented with noggin.	neurodevelopmental disorders, disease modeling,	[99]
human induced pluripotent stem cells	cornea, harboring three distinct cell types with the expression of key epithelial, stromal and endothelial cell markers.	multistep procotol	investigating corneal developmental processes and their disruptions in diseased condition	[100]
human embryonic stem cells (ESCs), human induced pluripotent stem cells (iPSCs)	3D ocular-like structures contains: retinal pigmented epithelium, neural retina, primitive lens and corneal-like structures.	differentiation: ventral neural induction media (VNIM) with IGF-1	development of eye, eye disorders, disease modeling,	[102]
human induced pluripotent stem cells (iPSCs)	3D multiocular organoids contans: retinal pigment epithelium, retina, and cornea.	multistep procotol	model the crosstalk between different cell types in eye development and disease	[104]
**Organs on Chip**				
Initial cells	Targeted cells	Culture techniques	Research possibilities	References
epithelium/stromal cells and keartnocytes isolated from rabbit eyes	microfluidic device consisting of a bilayer structure of corneal epithelial layer, a layer of stromal cells, and collagen vitrigel substrate	microfluidic devices containing collagen vitrigel (CV)	miniaturizing the standard transepithelial permeability (TEP) assay in order to measure the integrity of an array of corneal tissue micropatches.	[109]
immortalized human corneal epithelial cells	microengineered corneal epithelium-on-a-chip	porous membrane embedded microfluidic platform separated a chip into an apical and basal side	preclinical evaluations of potential therapeutic drugs and to mimic the environment of the human cornea.	[110]
human cells derived from the cornea and conjunctiva	mimic spontaneous eye blinking in humans	dome-shaped three-dimensional (3D) scaffolds in in vivo-like spatial arrangements	used for disease modeling and drug testing	[111]
human corneal epithelial (HCE–T) cells	dynamic cell cultivation and dynamic drug absorption testing on physiological barriers	DynaMiTES	improvement of common in vitro drug testing procedures	[112]

### 3.6. In Silico Analysis

In silico analysis is often used to meet the 3Rs regulations (replacement, reduction, and refinement) [114]. Many in silico models have been proposed up to this point in time. One of them is a quantitative structure-property relationship (QSPR) model proposed by Vincze et al. to study corneal permeability. The model is based on corneal-PAMPA (Parallel artificial membrane permeability assay) experimental data and different in silico drug transport parameters (Caco-2 and jejunal permeability) [115]. The test provided good predictions and is suitable for efficiently shortening the examined drugs list, provided we have comparable experimental data at our disposal. However, although promising, in silico studies currently do not provide us with enough data to regard drugs as safe. Therefore, experimental testing should be carried out to confirm the result of the studies.

## 4. Conclusions

Tissue engineering is one of the most rapidly developing scientific disciplines. It allows an easy and more reliable study of the effects of various factors and substances (including drugs). The development of this field will contribute to the invention of more advanced methods of combating diseases, repairing damaged tissues as a result of trauma, and to the ability to change and improve the function of given structures. At the same time, it will limit the number of animals used for experiments, which are now often indispensable research models. Currently, scientists are trying to fine-tune in vitro models and combine as many elements as possible to create a fully functional organ. One of the paths leading to this goal is the development of bioreactors. Bioreactors extend the time of in vitro culturing through specific, periodic exchanges of the culture medium. Physical factors are strictly controlled, e.g., temperature, pH, oxygen, and carbon dioxide. Additionally, they enable the precise delivery of nutrients and the removal of unnecessary metabolites from the nutrient solution [116,117].

All of the research models mentioned above have their limitations and advantages. Different Draize test alternative models provide more extensive flexibility in our research. Currently, 2D cultures are the most common research models. The reason is that 2D cultures are relatively inexpensive, more modulable, and easy to maintain [118]. Because of reproducible results obtained in controlled conditions [119], big-scale screening assays should be performed on these models. The main weakness of 2D models is their low ability to recreate the complexity of different cell classes and matrices interaction [118]. On the other hand, 3D multilayer models seem to be sufficient for small-scale drug toxicity and irritation assays. These models more closely resemble the eye microenvironment and consider cell-to-cell interactions, providing more relevant results. Moreover, 2D and 3D models seem to be limited in immunological disorders, such as allergy or sensitivity, because of their low of complexity. This problem could be addressed with organoids, which generate remarkable research outcomes, but only after long and arduous steps of standardization and testing [118].

Both in vitro and ex vivo models share one major limitation: the lack of vascularization [120]. The immune cells and vascularization should be introduced to these models to address this problem more appropriately. Organ-on-chip technology may be applied to facilitate the manipulation of more complex research models [120,121]. For example, including blood vessels in the model is possible by applying a forced flow supplied by on-chip technology. All that remains is to hope that the current development of in vitro models in ocular research allows for the complete elimination of the need to conduct tests on living organisms in the near future.

## Figures and Tables

**Table 1:** Layers of the eyeball and their functions.

Membrane	Part of Membrane	Structure and Function
Fibrous tunic	Sclera	✓ composed of collagen fibers ✓ surrounds 80% of the eye ✓ opaque ✓ protects against mechanical injuries ✓ place of muscle attachment ✓ maintains the oval shape of the eyeball
	Cornea	✓ avascular, consists of six layers ✓ surrounds 20% of the eyeball (from the front) ✓ transparent, permeable to light ✓ protects against mechanical injuries ✓ refracts the rays of light ✓ directs the light on the lens ✓ sensitive to pain ✓ the ability to regenerate from the limbus
Vascular anteriorly	Iris	✓ round, can change diameters ✓ around the pupil ✓ regulates the amount of light entering the inside of the eye ✓ contains a pigment in the epithelial layer on top of the muscular layers
	Ciliary body	✓ the muscle surrounding the lens ✓ changes the convexity of the lens (accommodation) ✓ connects the choroid with the iris ✓ responsible of the secretion of the aquous humor in the anterior chamber
	Choroid	✓ covers the retina ✓ consists of a dense network of capillaries of large diameter ✓ nourishes the cells of the eye ✓ it absorbs excess light
Nervous	Retina	✓ pigmented, photosensitive layer of the eye ✓ it determines the reception of visual impressions ✓ contains photosensitive cells (cone cells, rod cells) ✓ contains the macula of the retina and the blind spot (wherein the optic nerve head (ONH) is located)

## Data Availability

The data reviewed in this study are available on request from the corresponding author. The data are not publicly available due to founding agreement limitations.
